# Walking in School-Aged Children in a Dual-Task Paradigm Is Related to Age But Not to Cognition, Motor Behavior, Injuries, or Psychosocial Functioning

**DOI:** 10.3389/fpsyg.2016.00352

**Published:** 2016-03-10

**Authors:** Priska Hagmann-von Arx, Olivia Manicolo, Sakari Lemola, Alexander Grob

**Affiliations:** ^1^Department of Psychology, University of Basel, Basel, Switzerland; ^2^Department of Psychology, University of Warwick, Coventry, UK

**Keywords:** gait, dual-task walking, intelligence, executive functions, psychosocial functioning, school-aged children

## Abstract

Age-dependent gait characteristics and associations with cognition, motor behavior, injuries, and psychosocial functioning were investigated in 138 typically developing children aged 6.7–13.2 years (*M* = 10.0 years). Gait velocity, normalized velocity, and variability were measured using the walkway system GAITRite without an additional task (single task) and while performing a motor or cognitive task (dual task). Assessment of children’s cognition included tests for intelligence and executive functions; parents reported on their child’s motor behavior, injuries, and psychosocial functioning. Gait variability (an index of gait regularity) decreased with increasing age in both single- and dual-task walking. Dual-task gait decrements were stronger when children walked in the motor compared to the cognitive dual-task condition and decreased with increasing age in both dual-task conditions. Gait alterations from single- to dual-task conditions were not related to children’s cognition, motor behavior, injuries, or psychosocial functioning.

## Introduction

Walking is the most important mode of human locomotion (Adolph et al., 2003) and is a remarkably complex motor skill involving neural control systems that produce coordinated limb movements (Hausdorff, 2007). There is evidence that among typically developing children a mature gait pattern is established at about 7 years (Adolph et al., 2003). However, some studies investigating older children have shown that gait variability – subtle stride-to-stride fluctuations reflecting the regularity of a gait pattern – continues to develop across childhood into adolescence (e.g., Hausdorff et al., 1999).

Performing concurrent tasks, like listening to a conversation while walking, is an everyday behavior. Such dual-task situations adversely affect children’s walking, indicating that the regulation of gait requires cognitive processes such as executive and attentional functions (e.g., Cherng et al., 2007). Studies investigating children with developmental impairments have shown that children with motor and cognitive deficits are more vulnerable to dual-task gait decrements than typically developing children (e.g., Cherng et al., 2009; Katz-Leurer et al., 2011). Such decrements, in turn, may affect children’s motor behavior and psychosocial functioning, as it is known that children with poor motor skills participate in physical activity less frequently than controls (Cairney et al., 2005) and are at higher risk for peer rejection (Bejerot et al., 2013) as well as for social and emotional problems (Zwicker et al., 2012). However, these studies investigated children with developmental impairments, so it is still unclear if the maturity of walking patterns is related to cognition, motor behavior, and psychosocial functioning in typically developing children.

The aim of the present study was to shed light on age-dependent gait characteristics with a focus on gait variability in single- and dual-task walking and for the first time to examine the relation of dual-task effects on gait to cognition, motor behavior, and injuries, as well as psychosocial functioning in typically developing school-aged children.

After typically developing children master independent walking at approximately 12 to 14.5 months of age (Storvold et al., 2013), gait development is characterized by rapid improvements over the subsequent months (Bril and Ledebt, 1998; Adolph et al., 2003) until by the age of 3 years the visually apparent unsteadiness in walking has been replaced by a more stable gait pattern (Sutherland et al., 1988). With increasing age children show more subtle improvements in spatiotemporal gait parameters, including enhanced gait velocity and step length, and reach a mature gait pattern at about 7 years (e.g., Hillman et al., 2009; Holm et al., 2009). However, depending on the assessed gait parameters, there is evidence that gait continues to develop beyond this age (Hausdorff et al., 1999; Hillman et al., 2009; Lythgo et al., 2009, 2011; Froehle et al., 2013; Manicolo et al., 2016). For example, Hausdorff et al. (1999) investigated gait in typically developing children between 3 and 14 years of age. They measured gait velocity – considered a marker of general functional performance (cf. Al-Yahya et al., 2011) – as well as stride-to-stride fluctuations in spatiotemporal parameters (i.e., gait variability), which are sensitive to subtle physiological changes such as neural maturation and are believed to reflect the automaticity and regularity of gait (cf. Hausdorff, 2005). Results revealed that gait velocity was lowest in the youngest age group (aged 3 and 4 years) but did not significantly differ in the middle (aged 6 and 7 years) compared to the oldest (aged 11–14 years) age group, indicating a maturation of gait velocity at approximately 7 years of age. Results regarding gait variability showed a different picture. Gait variability continuously decreased from the youngest to the middle to the oldest age group. Thus, gait further developed after age 7 by becoming more automated and regular during middle and late childhood. In line with Hausdorff et al. (1999) findings, results of a recent study conducted by Abbruzzese et al. (2014) showed that when comparing gait variability of typically developing children aged 7–10 years to that of adults, children walked with significantly higher gait variability.

These findings indicate that among typically developing children gait variability decreases with age and that a mature gait pattern involving a high level of automaticity and regularity indicated by low gait variability may not be completely developed in school-aged children. Furthermore, these results highlight the sensitivity of gait variability measures and indicate their relevance when investigating children’s development of gait (Hausdorff, 2007).

In everyday life children usually do things concomitantly while walking, for example, listening to someone talk or fastening their jacket buttons. Such concurrent tasks may interfere with walking as less attention can be directed to the regulation of gait. Walking was for many years considered an automatic activity with only little involvement of cognitive processing. However, studies using a dual-task paradigm showed that gait is adversely affected when individuals are asked to walk and perform a concurrent task, indicating that walking requires cognitive resources (Huang and Mercer, 2001; Woollacott and Shumway-Cook, 2002; Huang et al., 2003; Cherng et al., 2007; Gill, 2015). Two theories have been used to explain dual-task effects on gait. The capacity-sharing theory proposes that attentional resources are limited in capacity and have to be shared between two tasks (Kahneman, 1973; Tombu and Jolicoeur, 2003). Researchers have suggested that gait decrements occur when the attentional demands of the concurrent task exceed the attentional resource capacity available (Huang and Mercer, 2001; Woollacott and Shumway-Cook, 2002). In contrast, the bottleneck theory (Pashler, 1994) proposes that two tasks that are performed simultaneously can only be carried out sequentially. This poses high demands on the capacity to switch between tasks, which in turn may lead to diminished performance in one or both of the tasks. However, there is currently no agreement on which theory best explains cognitive processing and dual-task effects (Yogev-Seligmann et al., 2008).

Studies investigating dual-task gait in typically developing children showed that performing a concurrent task while walking caused gait decrements (Huang et al., 2003; Cherng et al., 2007; Boonyong et al., 2012; Hung et al., 2013), indicating that also in children cognitive processes are involved when walking. Most studies on dual-task gait in children investigated spatiotemporal gait parameters such as velocity or stride length (e.g., Cherng et al., 2007; Boonyong et al., 2012; Hung et al., 2013), whereas only a few studies investigated gait variability. These revealed inconsistent results, with some researchers reporting no effect of dual tasking on gait variability (Leitner et al., 2007; Katz-Leurer et al., 2013; Abbruzzese et al., 2014) and others showing an increase in gait variability (Schaefer et al., 2010). However, the sample sizes in these studies were small, limiting the statistical power of these analyses.

Regarding age-dependent differences in dual-task gait, the effects of concurrent tasks on walking are stronger for younger compared to older typically developing children or adults. For example, Boonyong et al. (2012) examined the effect of an auditory concurrent task on spatiotemporal gait parameters in children aged 5 and 6 years, children aged 7–16 years, and healthy adults. Gait decrements such as reduced gait velocity in dual-task conditions were more profound in the younger compared to the older children and were greater in the two groups of children compared to the adults. Further, the study by Abbruzzese et al. (2014) revealed greater dual-task effects on gait variability in typically developing children aged 7–10 years compared to adults when they had to perform concurrent motor tasks such as carrying a tray with a pitcher, indicating that dual-task gait is still developing during childhood.

Furthermore, there is evidence that dual-task gait decrements are apparent for both motor and cognitive concurrent tasks. For instance, Cherng et al. (2007) investigated typically developing children aged 4–6 years while they walked and concurrently performed an easy or a difficult motor (carrying a tray with or without marbles on it) or cognitive (repeating a series of digits forward or backward) task. Results revealed that children showed poorer walking performance in the difficult motor task condition as well as in both cognitive task conditions compared to single-task walking. Comparing gait decrements caused by the different dual-task conditions revealed inconsistent results. The concurrent motor task led to greater decreases in stride length and greater increases in double limb support (i.e., the percentage of a gait cycle when both feet are on the ground) compared to the concurrent cognitive task. In contrast, the concurrent cognitive tasks led to greater increases in base of support (i.e., the area between the feet in contact with the ground), while effects on velocity and cadence (i.e., steps per minute) were not significantly different between task conditions. From a theoretical point of view the multiple-resource model of attention (Wickens, 1991) assumes that two tasks will interfere with each other if they share the same pool of resources. Therefore, one might expect that walking while performing a concurrent cognitive task might not cause the same level of gait decrements as a concurrent motor task that shares resources with walking (Yogev-Seligmann et al., 2008).

Studies investigating children with developmental impairments showed that dual-task effects on gait are stronger in children with poor motor skills (Cherng et al., 2009) and that also children with deficits in executive and attentional functions show more gait alterations while dual tasking than typically developing children. For example, children with severe post-traumatic brain injury showed reduced gait velocity as well as higher gait variability when they had to walk and concomitantly memorize and recall a series of numbers or listen to and identify commonly experienced sounds compared to typically developing children (Katz-Leurer et al., 2011). Further, children born very preterm walked with comparable gait velocity but higher stride velocity variability than their peers born at term when they had to listen to and memorize digits (Hagmann-von Arx et al., 2015). Similarly, studies investigating adults showed that dual-task effects on gait and particularly on gait variability are more profound in older individuals (Beurskens and Bock, 2012) and in patients with neurological impairments (Al-Yahya et al., 2011) who exhibit deficits in executive and attentional functions. Executive functions refer to higher cognitive processes that include the control and allocation of attentional resources necessary for adaptive planning of behaviors (Anderson, 2002). It is assumed that lower executive functions are associated with a reduced capacity to divide attention among multiple tasks and, therefore, go along with higher gait alterations in dual-task situations, as individuals are kept from devoting the appropriate attentional resources to their gait (Springer et al., 2006; Leitner et al., 2007). To our knowledge, however, no study has examined the relation between gait and cognition in typically developing school-aged children.

Motor performance affects other important aspects of children’s development (Piek et al., 2006b). Independent walking alters an infant’s relation to objects and people and allows the independent approach to new interaction partners (Iverson, 2010). These interactions, in turn, provide context for acquiring psychosocial skills (e.g., Karasik et al., 2011). However, motor skills that are not age-appropriately developed may negatively influence children’s behavior and psychosocial functioning. For instance, children with poor motor skills perform more poorly in individual as well as in team games and sports (Cantell et al., 1994; Skinner and Piek, 2001), which may lead them to voluntarily withdraw from situations in which they might demonstrate their motor abilities (Schoemaker and Kalverboer, 1994). In a similar vein, there is evidence that children with impaired motor coordination spend more time alone and participate less often in physical activity such as social play or organized sports than typically developing children (Cairney et al., 2005), which in turn places further motor development at risk (Bouffard et al., 1996). For example, there is evidence that children with low levels of physical activity have an increased risk of injury (Bloemers et al., 2012). Furthermore, children with motor deficits may be perceived by peers as being different or awkward, which may lead to peer rejection (Bejerot et al., 2013). For example, there is evidence that children with poor motor skills are at higher risk of being bullied at school than children with average motor skills (Piek et al., 2006a; Bejerot and Humble, 2013; Bejerot et al., 2013). Withdrawal from or exclusion by the peer group may lead to decreased self-esteem in children with poor motor skills, which in turn may increase emotional and behavioral problems such as symptoms of anxiety and depression (Zwicker et al., 2012).

Taken together, the evidence suggests that (a) in typically developing school-aged children gait is still developing in single and dual tasking, particularly regarding its regularity, (b) children with cognitive deficits show more gait decrements in dual-task conditions, and (c) poor motor skills are related to other aspects of children’s development. However, there are important gaps in the research. First, studies investigating age-dependent gait characteristics that include gait variability in single- and dual-task conditions in typically developing school-aged children are rare. Second, studies examining associations between dual-task gait effects and cognition in typically developing children are missing. Finally, to date, no study has examined the relation between dual-task gait effects, motor behavior, injuries, and psychosocial functioning in typically developing children.

In our study, we hypothesized the following: First, for single-task walking, we expected no association between age and gait velocity but hypothesized that age would be negatively related to gait variability, as the latter is sensitive to more subtle changes in gait (Hausdorff, 2005). Second, for dual-task walking, we hypothesized that age would be positively related to gait velocity and negatively related to gait variability, as there is evidence that dual-task gait is still developing (Boonyong et al., 2012; Abbruzzese et al., 2014). Third, we hypothesized that dual-task walking would lead to greater gait decrements (i.e., lower gait velocity and higher gait variability) compared to single-task walking, with greater gait decrements in a motor compared to a cognitive dual-task condition, drawing on the assumption that tasks sharing the same pool of processing resources interfere with each other more strongly (Yogev-Seligmann et al., 2008). Fourth, we hypothesized that cognitive and motor task performance would be decreased in dual- compared to single-task conditions, following the capacity-sharing theory (Kahneman, 1973; Tombu and Jolicoeur, 2003) as well as the bottleneck theory (Pashler, 1994) suggesting that dual-task walking may not only affect gait but also concurrent task performance. Finally, we hypothesized that less dual-task gait effects (i.e., lower change in gait velocity and gait variability from single- to dual-task walking) would be related to better cognitive performance, better motor behavior (i.e., higher sports participation) lower injury risk, and fewer injuries, as well as higher psychosocial functioning (i.e., higher physical and psychological well-being, better moods and emotions, higher self-perception and autonomy, better parent relation, more financial resources, better social support, better school environment, as well as higher social acceptance), as suggested by the notion that motor performance also affects other domains of children’s development (Piek et al., 2006b).

## Materials and Methods

### Participants

A total of 141 children (63 girls, 78 boys, *M*_age_ = 10.0 years, *SD* = 1.5, age range: 6.7–13.2 years) were recruited from birth announcements in newspapers as well as from local schools in the German-speaking part of Switzerland. All children were screened for developmental coordination disorder using the German version of the Movement Assessment Battery for Children (2nd ed.) with a cut-off below the 16th percentile (Petermann, 2008). Three children were excluded because of significant motor impairment. The final sample for this study consisted of 138 typically developing school-age children aged 6.7–13.2 years (*M* = 10.0 years, *SD* = 1.5; 62 girls, 76 boys) enrolled in public primary schools.

The local Ethics Committee approved the study. Parents gave written informed consent for the children to participate and assent was obtained from the children.

### Procedure

All children came to the laboratory for a visit that lasted approximately 3 h. Data were collected by trained study personnel. A battery of procedures was given in counterbalanced order to assess single- and dual-task gait and cognitive performance (i.e., intelligence, executive functions). In addition, children’s weight was measured with a digital scale, height was measured with a fixed stadiometer, and leg length was measured from greater trochanter to the floor, bisecting the lateral malleolus, with the children wearing their normal clothes and footwear. Parents completed questionnaires to provide information on demographic data, as well as children’s participation in sports, injury risk, and psychosocial functioning. Children received a gift voucher of CHF 30 for participating (1 CHF = 1.029 USD; February 2016) and parents received CHF 30 for completing the parental questionnaire.

### Measures

#### Gait Assessment

Gait parameters were measured using a portable GAITRite electronic walkway system (GAITRite Platinum; CIR Systems, USA). This walkway system consists of an electronic mat (length: 7.01 m, width: 0.9 m) with 23,040 integrated pressure sensors. To minimize the effects of acceleration and deceleration, two electronically inactive sections each with a length of 1.25 m were added on each end of the walkway system. Hence, each walk covered a distance of approximately 10 m with an average of eight steps. Children’s gait assessment using GAITRite has been established as reliable and valid (Thorpe et al., 2005). Gait analysis was performed according to European guidelines (Kressig and Beauchet, 2006). For each walk, the GAITRite software generates step-to-step values for a range of spatiotemporal gait parameters. The following gait parameters were derived: gait velocity which was measured in centimeters per second. In order to account for differences in children’s leg length we additionally normalized gait velocity to a dimensionless quantity using the formula suggested by Hof (1996, p. 223):

(1) $$normalized\,\;\;velocity\,\;\;=\frac{gait\,\;velocity}{\sqrt{\left(g\,\times l\right)}}$$

where *g* is the gravitational constant (9.81 m/s^2^) and *l* is leg length. Further, we assessed gait variability as stride-to-stride variability in stride velocity, stride time, and stride length all expressed as the percentage coefficient of variation (standard deviation/mean × 100).

Prior to gait assessment, children completed just the two tasks that would be used as the concurrent task for 10 s while standing so that their performance in these tasks could be determined. The concurrent tasks were selected according to related dual-task research. First, the children were asked to listen to and recall digits (digits task; Lindenberger et al., 2000; Leitner et al., 2007). For this task, children heard a list of randomized digits presented from a computer over loudspeakers that were installed at the front left and front right corner of the laboratory. Afterward, the children were asked to recall the digits. Second, children were asked to unfasten and fasten a button (button task) at stomach height (Ebersbach et al., 1995; Yang et al., 2007). Performance on this task was measured as the number of times the button could be unfastened and fastened.

Before the gait recordings, children were given one demonstration and one practice trial to familiarize them with the walkway system. Then, children were instructed to walk at their preferred pace without any additional task (single-task condition) with a total of four walking trials. Afterward, the children were instructed to walk at their preferred pace and to simultaneously perform one of the concurrent tasks (dual-task conditions) with two trials each. In the dual-task conditions children were not instructed to prioritize either one of the tasks. After each walk, gait data were analyzed using GAITRite software. For each child, gait parameters were averaged across the corresponding trials for further data analysis. All children successfully completed all walking trials in the first attempt. However, for two children (aged 7.6 and 8.1 years) dual-task gait parameters are not available because of technical error of GAITRite during the testing session.

#### Cognitive Functions

Intelligence was assessed using the German version of the Wechsler Intelligence Scale for Children (4th ed., WISC-IV; Petermann and Petermann, 2011). The WISC-IV is an individually administered instrument for assessing intellectual abilities in children and adolescents aged 6–16 years with established reliability and validity (e.g., Daseking et al., 2007; Hagmann-von Arx et al., 2012). The WISC-IV comprises 10 core subtests and five supplemental subtests, which were not administered in the current study. The subtests are assigned to four index scores (verbal comprehension, perceptual reasoning, working memory, processing speed) and are combined to form the full-scale IQ with a mean of 100 (*SD* = 15), representing a child’s global intellectual functioning. Due to restrictions in testing time intelligence scores are missing for six children.

Executive functions were measured using tasks from the computer-based Cambridge Neuropsychological Test Automated Battery (CANTAB touchscreen tests). CANTAB is suited for children aged from 4 years and provides highly reliable and valid measures for executive functions (e.g., Luciana, 2003). After a motor screening task, which introduced the CANTAB touchscreen to the children, they completed four tasks: The intra-extra dimensional (IED) set shift is a test of rule acquisition. It measures shifting and cognitive flexibility and records the number of errors made during the test. The rapid visual processing (RVP) test is a measure of vigilance or the ability to maintain a certain level of attention while engaged in a repetitive task. The outcome provides a measure of sensitivity to the target regardless of the response tendency. The stockings of Cambridge (SOC) task assesses the planning element of executive functions and records the number of problems solved in the minimum number of moves. Finally, the spatial working memory (SWM) test requires the child to maintain spatial information and to subsequently manipulate the presented items in working memory. The SWM task records the number of search errors. Scores reported in this study are standard scores based on age-corrected norms with *M* = 0 and *SD* = 1. Due to restrictions in testing time measures of executive functions are missing for six children.

#### Participation in Sports, Injury Risk, and Injuries

Parents reported whether their child was participating in sports. If yes, parents were further asked in which sport their child participated, how many times per week, and for how long. Scores for participation in sports reported in this study are number of minutes per week.

Parents also completed the German adaptation of the Injury Behavior Checklist (IBC; Brandau and Daghofer, 2010). The German IBC consists of 13 items regarding children’s risk-taking behaviors that can lead to injury. Parents are asked to rate the statements on a scale of 0 (*never*) to 3 (*very often*). The IBC has high reliability and established validity (Speltz et al., 1990; Brandau and Daghofer, 2010). Reliability in the present study was α = 0.76.

To assess injuries, we asked parents to report whether their child had been injured in the past 24 months. If yes, parents were further asked to list all injuries and provide information regarding the location of the accident, the activity being performed while injured, the type of injury, and whether the injury had to be treated. Scores for injuries reported in this study are number of injuries in the past 24 months. These parental reports are available for 129 children.

#### Psychosocial Functioning

Psychosocial functioning was assessed using the German version of the KIDSCREEN-52, a parental questionnaire with proven reliability and validity (Ravens-Sieberer et al., 2008). KIDSCREEN-52 consists of 52 items assessing the frequency of behavior and feelings or the intensity of an attitude using a 5-point Likert scale with the anchor points 1 (*never*) and 5 (*always*). The items are assigned to 10 dimensions: physical well-being, psychological well-being, moods and emotions, self-perception, autonomy, parent relation and home life, peers and social support, school environment, social acceptance/bullying, and financial resources. Reliability in the present study ranged from α = 0.67 (physical well-being) to α = 0.91 (psychological well-being). The KIDSCREEN-52 is available for 128 children.

### Statistical Procedure

Pearson’s correlations were computed to assess the relations between children’s demographic variables and all gait parameters in single- and dual-task conditions. Effects of age and dual-task conditions on gait were examined using repeated-measures multivariate analysis of variance (MANOVA) with one within-subject factor (walking condition: single-task vs. dual-task digits vs. dual-task button) and age as a continuous predictor. Significant effects were followed up with Bonferroni corrected *post hoc* pairwise comparisons. To assess effects of age on concurrent task performance, repeated-measures ANOVAs were performed separately for each walking condition with one within-subject factor (task performance: single task vs. dual task) and age as a continuous predictor. Extreme values in gait parameters defined as scores exceeding 3 *SD*s from the mean were truncated to ±3 *SD*. The level of significance was set to 0.05. The *F* statistic, *p*-values (two-tailed), and effect sizes (η^2^) as well as regression parameter estimates (standardized beta coefficients) for the relation of age to gait and concurrent task performance for each walking condition are reported.

To examine associations between gait and children’s cognition, motor behavior, and injuries, as well as psychosocial functioning we first calculated mean change values which are the mean differences between single-task and dual-task values. Positive signs denote a decrease from single-task to dual-task walking in the respective gait parameter, whereas negative signs denote an increase from single-task to dual-task walking in the respective gait parameter. Afterward, regression analyses with mean change values in gait parameters predicting children’s cognition, motor behavior, injuries, and psychosocial functioning were calculated controlling for age. Because of the high number of regressions performed, the level of significance was set to *p* < 0.01 to reduce the probability of alpha error accumulation. To estimate the statistical power given the sample size of the study, *post hoc* power analyses were performed using G^∗^Power (Faul et al., 2007). The chance of detecting medium-sized effects (*r* = 0.30) was 97% at a 0.01 alpha level. All analyses were performed using SPSS Statistics 22 for Apple Mac.

## Results

Means, standard deviations, and correlations among children’s demographic characteristics and gait parameters in single- and dual-task conditions are provided in **Table 1**.

**Table 1:** Descriptive statistics and correlations for demographics and gait parameters.

Variable		*M*	*SD*	Correlations																		
				1	2	3	4	5	6	7	8	9	10	11	12	13	14	15	16	17	18	19
**Demographics**											
1	Age	10.0	1.5	1																		
2	Sex (% male/female)^a^	(55/45)	–	–0.14	1																	
3	Height (cm)	141.7	10.6	0.79	–0.15	1																
4	Weight (kg)	35.0	8.6	0.68	–0.07	0.82	1															
5	Leg length (cm)	73.4	13.4	0.39	–0.09	0.55	0.47	1														
**Single task**
6	Velocity (cm/s)	132.6	19.0	–0.04	0.00	0.14	0.07	0.04	1													
7	Normalized velocity	4.9	0.7	–0.28	0.04	–0.20	–0.21	–0.27	0.94	1												
8	CV velocity (%)	2.6	0.9	–0.31	0.04	–0.28	–0.18	–0.20	–0.22	–0.12	1											
9	CV stride time (%)	1.9	0.6	–0.29	0.06	–0.30	–0.26	–0.18	–0.11	–0.01	0.57	1										
10	CV stride length (%)	2.1	0.7	–0.23	0.09	–0.28	–0.21	–0.24	–0.33	–0.23	0.71	0.40	1									
**Dual task: digits**
11	Velocity (cm/s)	105.3	16.0	0.08	0.04	0.10	–0.01	0.10	0.59	0.56	–0.23	–0.19	–0.31	1								
12	Normalized velocity	3.9	0.6	–0.14	0.08	–0.20	–0.25	–0.27	0.54	0.62	–0.14	–0.11	0.20	0.95	1							
13	CV velocity (%)	3.4	1.3	–0.18	–0.04	–0.15	–0.10	–0.01	–0.05	0.00	0.20	0.20	0.10	–0.29	–0.23	1						
14	CV stride time (%)	2.4	0.9	–0.21	–0.06	–0.20	–0.18	–0.08	–0.20	–0.13	0.33	0.32	0.25	–0.37	–0.29	0.64	1					
15	CV stride length (%)	2.4	1.0	–0.22	–0.12	–0.13	–0.07	–0.09	–0.08	–0.04	0.17	0.20	0.15	–0.36	–0.30	0.66	0.43	1				
**Dual task: button**
16	Velocity (cm/s)	88.1	16.9	0.23	–0.01	0.17	0.09	0.08	0.36	0.30	–0.19	–0.22	–0.31	0.66	0.59	–0.28	–0.35	–0.30	1			
17	Normalized velocity	3.2	0.6	0.05	0.01	–0.07	–0.09	–0.07	0.36	0.38	–0.12	–0.15	–0.24	0.68	0.68	–0.24	–0.31	–0.26	0.96	1		
18	CV velocity (%)	5.1	2.3	–0.31	0.02	–0.14	0.00	–0.04	0.11	0.14	0.16	0.12	0.13	–0.25	–0.20	0.20	0.22	0.25	–0.59	–0.56	1	
19	CV stride time (%)	3.5	1.6	–0.22	0.00	–0.08	0.02	–0.06	0.03	0.06	0.16	0.13	0.17	–0.31	–0.28	0.14	0.31	0.23	–0.60	–0.59	0.77	1
20	CV stride length (%)	3.7	2.0	–0.28	–0.05	–0.16	–0.07	–0.11	0.00	0.07	0.18	0.12	0.24	–0.29	–0.22	0.12	0.27	0.27	0.55	–0.52	0.78	0.71


*CV, coefficient of variation*. ^a^*0 = male, 1 = female; Significance*: *p* < 0.05: *r* > 0.17; *p* < 0.01: *r* > 0.22; *p* < 0.001: *r* > 0.27.

### Age Effects on Gait in Single- and Dual-Task Conditions

**FIGURE 1:**
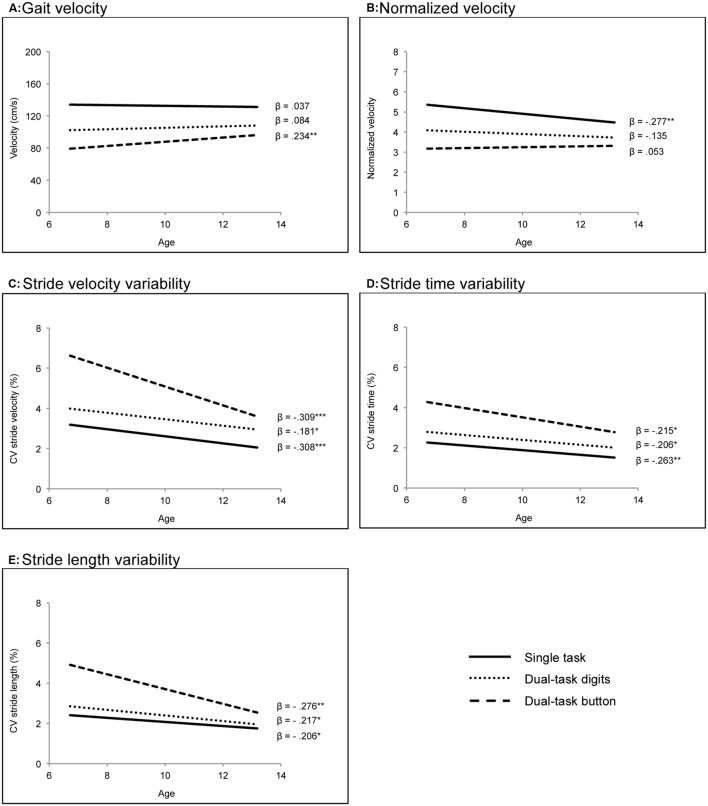
**Associations of age (in years) with gait velocity **(A)**, normalized velocity **(B)**, stride velocity variability **(C)**, stride time variability **(D)**, and stride length variability **(E)** in single-task walking and in the dual-task conditions digits and button.** CV, coefficient of variation. ^∗^*p* < 0.05, ^∗∗^*p* < 0.01, ^∗∗∗^*p* < 0.001.

Repeated-measures MANOVAs were used to analyze the effects of dual tasking and age on gait. Regarding gait velocity, results revealed a significant within-subject effect of walking condition (Wilks’s multivariate test), *F*(2,133) = 21.154, *p* < 0.001, η^2^= 0.241. Pairwise comparisons revealed higher gait velocity in single-task walking compared to both dual-task conditions (*p* < 0.001) and higher gait velocity in the dual-task condition digits compared to button (*p* < 0.001). There was no significant between-subjects effect of age but a significant Walking Condition × Age interaction (Wilks’s multivariate test), *F*(2,133) = 3.956, *p* = 0.021, η^2^= 0.056: While the effect of age was not significant for single-task walking or the dual-task condition digits, it was significant for the dual-task condition button, such that older children walked with higher gait velocity than younger children when unfastening and fastening a button. The regression parameter estimates for the associations between age and gait velocity in each walking condition are depicted in **Figure 1A**.

Regarding normalized velocity, results revealed a significant within-subject effect of walking condition (Wilks’s multivariate test), *F*(2,130) = 28.720, *p* < 0.001, η^2^= 0.306. Pairwise comparisons revealed higher normalized velocity in single-task walking compared to both dual-task conditions (*p* < 0.001) and higher normalized velocity in the dual-task condition digits compared to button (*p* < 0.001). The between-subjects effect of age was marginally significant, *F*(1,131) = 3.141, *p* = 0.079, η^2^= 0.023, such that older children walked with lower normalized velocity than younger children. Further, the Walking Condition × Age interaction was significant (Wilks’s multivariate test), *F*(2,130) = 7.192, *p* < 0.001, η^2^= 0.100, indicating that the effect of age on normalized velocity was stronger in the single-task condition compared to the dual-task conditions. The regression parameter estimates for the associations between age and normalized velocity in each walking condition are depicted in **Figure 1B**.

For stride velocity variability, repeated-measures MANOVAs revealed a significant within-subject effect of walking condition (Wilks’s multivariate test), *F*(2,133) = 8.229, *p* < 0.001, η^2^= 0.110. Pairwise comparisons revealed lower variability in single-task walking compared to both dual-task walking conditions (*p* < 0.001) and lower variability in the dual-task condition digits compared to button (*p* < 0.001). Furthermore, there was a significant between-subjects effect of age, *F*(1,134) = 22.990, *p* < 0.001, η^2^= 0.146, such that older children walked with lower variability than younger children in all walking conditions. The Walking Condition × Age interaction was marginally significant (Wilks’s multivariate test), *F*(2,133) = 2.800, *p* = 0.064, η^2^= 0.040, indicating that the effect of age on gait variability tended to be stronger in the single-task condition and dual-task condition button compared to the dual-task condition digits. The regression parameter estimates for the associations between age and stride velocity variability in each walking condition are depicted in **Figure 1C**.

Regarding stride time variability, results revealed a significant within-subject effect of walking condition (Wilks’s multivariate test), *F*(2,133) = 4.546, *p* = 0.012, η^2^= 0.064. Pairwise comparisons revealed lower variability in single-task walking compared to both dual-task conditions (*p* < 0.001) and lower variability in the dual-task condition digits compared to button (*p* < 0.001). Furthermore, there was a significant between-subjects effect of age, *F*(1,134) = 13.328, *p* < 0.001, η^2^= 0.090, such that older children walked with lower gait variability than younger children. There was no significant Walking Condition × Age interaction. The regression parameter estimates for the associations between age and stride time variability in each walking condition are shown in **Figure 1D**.

Regarding stride length variability, results revealed a significant within-subject effect of walking condition (Wilks’s multivariate test), *F*(2,133) = 7.690, *p* = 0.001, η^2^= 0.104. Pairwise comparisons revealed lower variability in single-task walking compared to both dual-task conditions (*p* < 0.003) and lower variability in the dual-task condition digits compared to button (*p* < 0.001). Furthermore, there was a significant between-subjects effect of age, *F*(1,134) = 16.819, *p* < 0.001, η^2^= 0.112, such that older children walked with lower gait variability than younger children. Finally, there was a significant Walking Condition × Age interaction (Wilks’s multivariate test), *F*(2,133) = 3.114, *p* = 0.048, η^2^= 0.045, such that the effect of age on gait variability was stronger in the dual-task condition button compared to single-task walking and the dual-task condition digits. The regression parameter estimates for the associations between age and stride length variability in each walking condition are shown in **Figure 1E**.

### Age Effects on Concurrent Task Performance in Single- and Dual-Task Conditions

Children’s performances of recalling digits in the single-task (*M* = 3.94, *SD* = 0.89) and dual-task (*M* = 4.10, *SD* = 0.91) conditions were comparable, *F*(1,133) = 1.277, *p* = 0.260, η^2^= 0.010. However, children’s performance increased with age, *F*(1,133) = 12.674, *p* < 0.001, η^2^= 0.087; Regression parameter estimates were β = 0.295, *p* < 0.001 for the single-task condition and β = 0.220, *p* = 0.010 for the dual-task condition. There was no significant Task Performance × Age interaction, *F*(1,133) = 0.702, *p* = 0.403, η^2^= 0.005.

Children’s performances of unfastening and fastening a button in the single-task (*M* = 5.16, *SD* = 1.67) and dual-task (*M* = 6.31, *SD* = 1.90) conditions were not significantly different, *F*(1,132) = 2.509, *p* = 0.116, η^2^= 0.019, and were comparable across age [age: *F*(1,132) = 3.524, *p* = 0.063, η^2^= 0.026; interaction: *F*(1,132) = 0.159, *p* = 0.691, η^2^= 0.001]; regression parameter estimates were β = 0.171, *p* = 0.050 for the single-task condition and β = 0.119, *p* = 0.172 for the dual-task condition.

### Gait and Associations with Cognition, Motor Behavior, and Injuries, as well as Psychosocial Functioning

Means and standard deviations of children’s cognitive performance, motor behavior, and injuries, as well as psychosocial functioning are presented in **Table 2**.

**Table 2:** Means and standard deviations of children’s cognition, motor behavior and injuries, as well as psychosocial functioning.

Variable	*n*	*M*	*SD*	Range
**Cognition**				
Intelligence (WISC-IV FSIQ)	132	105.94	10.48	84–136
Executive functions (CANTAB)				
IED	131	0.24	1.04	–6.64–1.89
RVP	127	-0.09	1.23	–4.96–1.25
SOC	132	0.01	0.83	–2.61–1.66
SWM	131	0.38	0.89	–2.22–2.75
**Motor behavior and injuries**				
Participation in sports (minutes/week)	129	214.98	153.51	0–870
Injury risk (IBC)	129	6.20	3.84	0–21
Injuries (number)	127	0.97	1.17	0–5
**Psychosocial functioning (KIDSCREEN-52)**				
Physical well-being	125	20.91	2.95	12.50–25
Psychological well-being	126	26.01	3.41	14–30
Moods and emotions	126	11.47	3.45	7–23
Self-perception	126	22.30	2.52	12–25
Autonomy	127	20.75	2.61	13–25
Parent relation and home life	127	25.28	3.06	17–30
Financial resources	125	13.30	2.28	3–15
Social support and peers	127	25.16	3.78	8–30
School environment	128	25.91	3.43	12–30
Social acceptance (bullying)	128	4.40	1.68	3–10


*CANTAB, Cambridge Neuropsychological Test Automated Battery; IBC, injury behavior checklist; IED, intra-extra dimensional set shift; RVP, rapid visual processing; SOC, stockings of Cambridge; SWM, spatial working memory; WISC-IV FSIQ, Wechsler Intelligence Scales for Children 4th ed., full-scale IQ*.

Mean change values between single-task and dual-task gait parameters are presented in **Table 3**. The mean change values were positive for both velocity parameters, indicating a decrease in gait velocity and normalized velocity from single- to dual-task walking, whereas the mean change values were negative for all gait variability parameters, indicating an increase in gait variability from single- to dual-task walking. Regression analyses were calculated to examine the relation between mean change values in gait parameters and children’s cognition, motor behavior, and injuries, as well as psychosocial functioning. Results are shown in **Table 3**. Controlling for age, there were no significant associations between mean change values in gait parameters and other aspects of children’s development (all *p* ≥ 0.01).

**Table 3:** Regression analyses with mean changes in gait predicting children’s cognition, motor behavior and injuries, as well as psychosocial functioning controlled for age.

Mean changes			Cognition					Motor behavior and injuries			Psychosocial functioning									
	*M*	*SD*	FSIQ	IED	RVP	SOC	SWM	Sports	IBC	Injuries	PHW	PSW	ME	SP	AUT	PRHL	FR	SSP	SE	SA
**Dual task: Digits**																				
Velocity	27.3	16.2	0.01	0.04	–0.15	–0.05	0.07	0.03	0.10	0.06	0.09	0.02	–0.01	0.10	–0.03	0.03	–0.06	0.02	0.02	–0.10
Normalized velocity	1.0	0.6	–0.01	0.06	–0.14	–0.06	0.10	0.02	0.11	0.04	0.08	0.01	0.00	0.08	–0.03	0.03	–0.09	0.00	0.00	–0.08
CV velocity	–0.8	1.4	0.03	0.04	–0.03	0.16	–0.10	0.00	–0.13	–0.11	–0.03	0.06	–0.10	–0.01	0.14	0.03	0.09	–0.03	0.12	–0.04
CV stride time	–0.3	1.1	0.02	–0.02	0.05	0.08	–0.08	–0.17	–0.13	–0.05	–0.09	–0.20	0.05	–0.04	0.09	–0.04	–0.01	–0.11	0.13	–0.03
CV stride length	–0.5	0.9	0.05	0.07	–0.11	0.11	–0.04	–0.06	–0.06	0.03	0.13	0.23	–0.13	0.07	0.17	0.21	0.19	0.05	0.19	–0.07
**Dual task: Button**																				
Velocity	44.5	20.4	0.01	0.00	–0.06	–0.16	0.00	–0.03	–0.13	0.05	–0.01	–0.04	0.07	0.07	–0.05	0.05	–0.03	0.03	0.11	–0.05
Normalized velocity	1.7	0.8	0.01	0.01	–0.06	–0.18	–0.01	–0.05	–0.10	0.05	0.00	–0.11	0.12	–0.02	–0.04	0.02	–0.05	0.01	0.05	–0.03
CV velocity	–2.5	2.3	0.07	0.07	0.06	0.10	0.02	0.08	0.06	0.09	0.10	0.10	0.16	0.05	0.03	0.03	–0.08	–0.07	0.23	–0.09
CV stride time	–1.6	2.0	–0.03	0.00	0.08	0.15	0.05	–0.04	0.03	0.06	0.09	0.13	–0.11	0.03	–0.02	0.02	–0.09	–0.07	0.15	–0.19
CV stride length	–1.6	1.7	0.07	0.04	–0.04	0.14	0.11	–0.02	–0.06	0.11	0.16	0.12	–0.15	0.07	–0.02	0.10	0.00	–0.05	0.17	–0.19


*Coefficients are standardized regression coefficients if not otherwise indicated. FSIQ, full-scale IQ; IED, intra-extra dimensional set shift; RVP, rapid visual processing; SOC, stockings of Cambridge; SWM, spatial working memory; IBC, injury behavior checklist; PHW, physical well-being; PSW, psychological well-being; ME, moods and emotions; SP, self-perception; PRHL, parent relation and home life; FR, financial resources; SSP, social support and peers; SE, school environment; SA, social acceptance (bullying); CV, coefficient of variation. All *p* ≥ 0.01.*

## Discussion

The aim of the present study was to investigate age-dependent gait characteristics in single- and dual-task walking and to examine the relation of gait to cognition, motor behavior, and injuries, as well as psychosocial functioning for the first time in typically developing school-aged children. In single-task walking the present study revealed no association between age and gait velocity. This is in accordance with previous research, indicating a maturation of gait velocity at the age of 6–7 years (Hausdorff et al., 1999). However, a negative relation was found between age and normalized velocity. Thus, when accounting for differences in leg length of the children, which were correlated with age and therefore may have confounded our results, older children showed lower velocity than younger children. This finding contradicts previous results where normalized gait velocity was unaffected by age (Dusing and Thorpe, 2007). However, in our study velocity was normalized to leg length (Hof, 1996) whereas Dusing and Thorpe (2007) normalized data to height. Hence, due to the differing methods of normalization, it may not be possible to directly compare the results.

Further, a negative relation was found between age and gait variability what is in line with previous research showing higher gait variability in younger compared to older typically developing school-aged children (Hausdorff et al., 1999). These results provide evidence that gait variability, which is sensitive to more subtle physiological changes such as neural maturation than spatiotemporal gait parameters (Hausdorff, 2005), continues to develop across middle childhood into adolescence. Further, our results highlight the importance of not only assessing spatiotemporal gait parameters but also considering gait variability as an index of gait automaticity and regularity when investigating typically developing children’s gait maturation, because these gait measures seem to undergo temporally distinct developmental trajectories.

In dual-task conditions, where children were asked to walk and simultaneously perform a concurrent cognitive task (i.e., listening to and memorizing digits) or motor task (i.e., unfastening and fastening a button), children walked with reduced gait velocity and normalized gait as well as increased gait variability compared to single-task walking. These results are in line with previous research showing that dual-task walking leads to gait decrements in spatiotemporal gait parameters (Cherng et al., 2007; Boonyong et al., 2012; Hung et al., 2013) as well as in gait variability measures (Schaefer et al., 2010), although other studies showed no effect of dual-task walking on gait variability in typically developing children (Leitner et al., 2007; Katz-Leurer et al., 2013; Abbruzzese et al., 2014). Our result that gait was adversely affected when children were asked to walk and perform a concurrent task supports the notion that gait requires cognitive resources (Huang and Mercer, 2001; Woollacott and Shumway-Cook, 2002; Huang et al., 2003; Cherng et al., 2007). However, the underlying mechanisms for the here reported dual-task interference are not clear (Yogev-Seligmann et al., 2008). In accordance with the capacity-sharing theory (Kahneman, 1973; Tombu and Jolicoeur, 2003), it is possible that having to share limited attentional resources between two attention-demanding tasks lead to decreased task performance in one or both of the tasks. On the other hand, following the bottleneck theory (Pashler, 1994), which claims that two simultaneously performed tasks are cognitively processed sequentially, it may be that switching from one task to the other leads to diminished performance in one or both of the tasks. Therefore, we not only investigated whether gait parameters changed from single- to dual-task walking but also whether the concurrent task performance differed between single- and dual-task conditions. Results showed that, while gait parameters significantly changed from single- to dual-task walking, concurrent task performance (i.e., number of recalled digits and number of times a button could be unfastened and fastened) did not differ between single- and dual-task conditions. Hence, although children were not instructed to prioritize one task over the other, they possibly followed a “posture second” strategy (Bloem et al., 2006) by prioritizing the concurrent task over their walking performance.

Regarding age-dependent dual-task effects on gait, the results revealed that in the motor dual-task condition, age was positively related to gait velocity. This result is in line with previous research on typically developing children showing that younger children walked with lower gait velocity than older children when concurrently performing a second task (Boonyong et al., 2012). However, in our study there were no age-dependent dual-task effects on gait velocity in the cognitive dual-task condition. Normalized velocity showed no age-dependent dual-task effects. Further, in both dual-task conditions, age was negatively related to gait variability such that younger children walked with higher gait variability than older children when concurrently listening to and memorizing digits or unfastening and fastening a button. These results are in line with the study conducted by Abbruzzese et al. (2014) and indicate that gait in dual-task conditions is still developing in middle childhood.

Our results further show that the dual-task effects on walking differed between the two types of concurrent tasks: When walking and concurrently unfastening and fastening a button, children showed greater decrease in gait velocity and normalized velocity, as well as a greater increase in gait variability compared to when walking and concurrently listening to and memorizing digits. This finding indicates that a concurrent motor task may lead to greater dual-task gait decrements than a concurrent cognitive task and can be interpreted from the perspective of the multiple-resource model of attention (Wickens, 1991; Cherng et al., 2007). This model assumes that attentional resources are not unitary but are divided into various pools, which, for example, depend on the modality of input and response. Walking requires visual input and further involves the response of moving and controlling body segments, which Cherng et al. (2007) subsumed under the term somatosensation. The motor concurrent task of unfastening and fastening a button also requires visual input as well as somatosensory response. In contrast, the cognitive concurrent task of listening to and recalling digits requires auditory input and involves vocal response. According to these assumptions, the motor dual-task competes more strongly for processing resources with walking (i.e., visual input and somatosensory response) than the cognitive dual-task, which may have led to greater dual-task gait decrements in the motor compared to the cognitive dual-task condition.

Further, performance in the concurrent cognitive task of listening to and memorizing digits was associated with age such that older children recalled more digits than younger children. This is in accordance with previous research showing that younger children score lower than older children in working memory tests (Gathercole et al., 2004). Performance in the motor task of unfastening and fastening a button, however, was not related to age.

Finally, we investigated whether change in gait from single- to dual-task conditions (i.e., dual-task gait effects) is associated with cognition and other aspects of children’s development. Our results revealed no significant relations. Thus, we conclude that contrary to our hypothesis, dual-task gait effects were not meaningfully related to children’s cognition, motor behavior, and injuries, or psychosocial functioning. It has to be noted that our hypotheses were derived from studies comparing individuals with cognitive and motor impairments to typically developing controls. For example, regarding cognition, there is evidence that children and adults with deficits in executive and attentional functions show more gait alterations in single- and dual-task conditions compared to controls (e.g., Al-Yahya et al., 2011; Katz-Leurer et al., 2013). Regarding motor behavior and injuries, previous research investigating children with poor motor skills showed that these children participate less often in organized sports (Cairney et al., 2005) and have an increased risk of injury (Bloemers et al., 2012) compared to controls. Regarding psychosocial functioning, such as social acceptance or psychological well-being, previous findings investigating children with poor motor skills showed that these children are at higher risk for being bullied at school (Piek et al., 2006a; Bejerot and Humble, 2013; Bejerot et al., 2013) or for showing symptoms of anxiety and depression (Zwicker et al., 2012). However, we are not aware of studies investigating direct relations between gait and cognition, motor behavior and injuries, and psychosocial functioning in typically developing children. Therefore, our study is the first to provide preliminary evidence that dual-task effects in gait velocity, normalized velocity, and gait variability are not related to these aforementioned aspects of child development during middle childhood. However, the dual-task gait decrements apparent in our study support the notion that also among typically developing children, cognitive processes play an important role in gait. Hence, future studies might investigate whether other cognitive processes that were not investigated in this study, such as inhibition (i.e., inhibiting a prepotent reaction in favor of a less automated response) or cognitive flexibility (i.e., directing the attentional focus from one task to another; Miyake et al., 2000), also contribute to gait performance of typically developing children.

Our study has strengths and limitations. We consider it a strength that gait characteristics were assessed using the GAITRite system, which has proved to be a valid method of measuring gait parameters in children and offers the possibility of reliably identifying subtle changes in gait (Thorpe et al., 2005). During gait assessment children wore their normal clothes and shoes and it was therefore possible to assess gait performance as it is exhibited under everyday circumstances. However, although we investigated age-dependent gait characteristics in single- and dual-task walking of school-aged children, it was not possible to determine at what age gait characteristics, which are still developing during middle childhood, reach maturity, as we did not investigate a comparison sample of adult participants. Furthermore, the children in our study were first asked to perform single-tasks followed by dual-tasks. Therefore, we cannot rule out the possibility that a practice effect benefited the concurrent task performance while dual-tasking. In order to further investigate practice effects, future studies might apply task conditions in counter-balanced order or they might include a control group, which repeats the tasks only in single-task conditions. Additionally, our analyses were performed on cross-sectional data, whereas the testing of developmental trends in single- and dual-task walking should include longitudinal data in future investigations. Future research might also include different types of concurrent tasks when investigating children’s gait in dual-task conditions because previous research also showed interference effects on gait for visual and auditory concurrent tasks among typically developing children (Huang et al., 2003). Finally, we investigated gait in straight walking. Future studies might examine age-dependent gait characteristics of walking along curved trajectories (Belmonti et al., 2013), as curvilinear walking may be more common in our everyday life.

## Conclusion

This study provides important information on age-related changes in gait during middle childhood. Our findings indicate that gait in typically developing children becomes more regular with increasing age in single- and dual-task walking, thereby highlighting the importance of including measures of gait variability when investigating gait development. Since we found dual-task gait decrements to be larger when walking and concurrently performing a motor compared to a cognitive task, our results underscore the importance of taking the type of concurrent task into account when investigating children’s gait in a dual-task paradigm. Finally, our study revealed no association of dual-task gait effects with children’s cognition, motor behavior and injuries, or psychosocial functioning, indicating that subtle dual-task effects on gait do not go along with other aspects of development in typically developing children during middle childhood.

## Author Contributions

PH and OM contributed to the study design, acquisition, analysis and interpretation of data. Drafted and revised the manuscript, gave final approval, and agree to be accountable for all aspects of the work in ensuring that questions related to the accuracy or integrity of any part of the work are appropriately investigated and resolved. Both authors contributed the same amount of work to this paper. SL contributed to the analysis and interpretation of data, revised the manuscript, gave final approval, and agrees to be accountable for all aspects of the work in ensuring that questions related to the accuracy or integrity of any part of the work are appropriately investigated and resolved. AG contributed to the study design and interpretation of data, revised the manuscript, gave final approval, and agrees to be accountable for all aspects of the work in ensuring that questions related to the accuracy or integrity of any part of the work are appropriately investigated and resolved.

## Conflict of Interest Statement

The authors declare that the research was conducted in the absence of any commercial or financial relationships that could be construed as a potential conflict of interest.
